# Characterization of Extensively Drug-Resistant Salmonella enterica Serovar Kentucky Sequence Type 198 Isolates from Chicken Meat Products in Xuancheng, China

**DOI:** 10.1128/spectrum.03219-22

**Published:** 2023-02-27

**Authors:** Yue Jiang, Zhen-Yu Wang, Qiu-Chun Li, Meng-Jun Lu, Han Wu, Cai-Yue Mei, Peng-Cheng Shen, Xinan Jiao, Jing Wang

**Affiliations:** a Jiangsu Key Laboratory of Zoonosis/Jiangsu Co-Innovation Center for Prevention and Control of Important Animal Infectious Diseases and Zoonoses, Yangzhou University, Yangzhou, China; b Key Laboratory of Prevention and Control of Biological Hazard Factors (Animal Origin) for Agrifood Safety and Quality, Ministry of Agriculture of China, Yangzhou University, Yangzhou, China; University of Maryland Eastern Shore

**Keywords:** *bla*_CTX-M-55_, chromosome, *Salmonella*, ST198, *Salmonella* genomic island

## Abstract

The purpose of this study was to characterize extensively drug-resistant Salmonella enterica serovar Kentucky sequence type 198 (ST198) isolates from chicken meat products. Ten *S*. Kentucky strains obtained from chicken meat products in Xuancheng, China, carried 12 to 17 resistance genes, such as *bla*_CTX-M-55_, *rmtB*, *tet*(A), *floR*, and *fosA3*, combined with mutations within *gyrA* (S83F and D87N) and *parC* (S80I), resulting in resistance to numerous antimicrobial agents, including the clinically important antibiotics cephalosporin, ciprofloxacin, tigecycline, and fosfomycin. These *S*. Kentucky isolates shared a close phylogenetic relationship (21 to 36 single-nucleotide polymorphisms [SNPs]) and showed close genetic relatedness to two human clinical isolates from China. Three *S*. Kentucky strains were subjected to whole-genome sequencing using Pacific Biosciences (PacBio) single-molecule real-time (SMRT) technology. All antimicrobial resistance genes were located on their chromosomes and clustered in one multiresistance region (MRR) and Salmonella genomic island (SGI) SGI1-K. The MRRs in three *S*. Kentucky strains were bounded by IS*26* at both ends and were inserted downstream of the *bcfABCDEFG* cluster with 8-bp direct repeats. The MRRs were related to those of IncHI2 plasmids but differed by insertions, deletions, and rearrangements of multiple segments involving resistance genes and plasmid backbones. This finding suggests that the MRR fragment possibly originates from IncHI2 plasmids. Four SGI1-K variants with slight differences were identified in 10 *S*. Kentucky strains. Mobile elements, particularly IS*26*, play an essential role in forming distinct MRRs and SGI1-K structures. In conclusion, the emergence of extensively drug-resistant *S*. Kentucky ST198 strains containing numerous chromosomally located resistance genes is alarming and needs continued surveillance.

**IMPORTANCE** Salmonella spp. are important foodborne pathogens, and multidrug-resistant (MDR) Salmonella strains have become a serious threat to clinical therapy. MDR *S*. Kentucky ST198 strains have been increasingly reported from various sources and have become a global risk. In this study, we described extensively drug-resistant *S*. Kentucky ST198 strains from chicken meat products from a city in China. Numerous resistance genes are clustered in the chromosomes of *S*. Kentucky ST198 strains, possibly acquired with the help of mobile elements. This would facilitate the spread of numerous resistance genes as intrinsic chromosomal genes within this global epidemic clone, with the potential to capture more resistance genes. The emergence and dissemination of extensively drug-resistant *S*. Kentucky ST198 pose a severe clinical and public health threat; therefore, continuous surveillance is warranted.

## INTRODUCTION

Salmonella is one of the most common foodborne pathogens causing human gastroenteritis and is often associated with the contamination of poultry products (1, 2). In 2018, salmonellosis was the second most common zoonosis, with 91,857 human cases, and was the most frequent cause of foodborne outbreaks in the European Union (3, 4). Third-generation cephalosporins and fluoroquinolones are the highest-priority clinically important agents for treating severe human salmonellosis (5). However, resistance to cephalosporins and fluoroquinolones in Salmonella has been increasingly reported (2, 4). Worse still, multidrug-resistant (MDR) Salmonella strains have emerged and been widely disseminated and have become a significant challenge for clinical therapy (2, 4).

Salmonella enterica serovar Kentucky has become a common nontyphoid Salmonella serotype associated with human infection (4). Before the 1990s, this serotype was mainly associated with poultry and was susceptible to all antibiotics (6). The MDR *S*. Kentucky sequence type 198 (ST198) emerged due to the chromosomal integration of Salmonella genomic island (SGI) SGI1-K, probably in the mid-1990s (6). SGI1 is a mobilizable element that was first identified in the Salmonella enterica serovar Typhimurium strain DT104 and carries five resistance genes, conferring resistance to multiple antimicrobial agents (7). In the early 2000s, *S.* Kentucky ST198 clones accumulated three amino acid substitutions in GyrA and ParC, leading to high-level resistance to ciprofloxacin (6, 8). Since then, the ciprofloxacin-resistant *S*. Kentucky ST198 has rapidly spread around the world, mainly in Europe, North Africa, the Middle East, and South Asia, and has become highly drug resistant through the acquisition of extended-spectrum β-lactamase (CTX-M), cephalosporinase (CMY), or carbapenemase (OXA-48, VIM, and NDM) genes (6, 8, 9). MDR *S*. Kentucky ST198 has become a global epidemic clone, and domestic poultry is an important vehicle for its global spread (6, 9). However, *S*. Kentucky ST198 has been sporadically reported in China. A low prevalence (0.18% [27/15,405 strains]) of *S*. Kentucky ST198 was identified among S. enterica strains from patients, poultry, and meat products in China in 2013 to 2017 (10). Meanwhile, a large proportion (60.3%) of ciprofloxacin resistance was observed among *S*. Kentucky ST198 strains from human, environmental, and chicken samples in 2010 to 2016 (11). MDR *S*. Kentucky ST198 has been sporadically reported in broiler chickens, poultry meat, slaughterhouse, and vegetable in China (12–15). In this study, we investigated the characterization of extensively drug-resistant *S*. Kentucky ST198 isolates from chicken meat products from supermarkets and a slaughterhouse in a city of Anhui Province, China, to elucidate the genetic basis for extensively drug-resistant *S*. Kentucky ST198.

## RESULTS AND DISCUSSION

### Characterization of Salmonella isolates detected in food samples.

A total of 13 Salmonella strains (17.3%) were isolated from 75 chicken meat and internal organ samples. Three serotypes were identified among 13 Salmonella strains. *S.* Kentucky (*n* = 10) was the most prevalent serotype, and all of the *S.* Kentucky strains belonged to ST198. Salmonella enterica serovar Agona ST13 (*n* = 2) and Salmonella enterica serovar Infantis ST32 (*n* = 1) were also detected. The high prevalence of *S*. Kentucky ST198 (13.33% [10/75 strains]) in this study is noteworthy; it was much higher than those in the chicken supply chain (0.54% [23/4,236 strains]) and among patients (0.33% [40/12,011 strains]) in China between 2010 and 2016 (11). However, the small number of samples in a single city is a limitation of this study. Recently, *S*. Kentucky ST198 became the second most prevalent serotype (3.10% [13/420 strains]) in the production chain of broiler chickens in Sichuan Province, China (13). It has also been increasingly detected in animals and clinics in countries outside China, e.g., India (3.49% in animals and 0.31% in patients), East Africa, Spain, and Switzerland (16–19).

All 13 Salmonella isolates were tested for the MICs of 15 antimicrobial agents. They were all susceptible to meropenem and colistin. Additionally, 10 *S*. Kentucky strains were resistant to ampicillin, cefazolin, cefotaxime, gentamicin, streptomycin, amikacin, tetracycline, tigecycline, chloramphenicol, florfenicol, nalidixic acid, ciprofloxacin, and sulfamethoxazole-trimethoprim, and 8 of them also exhibited resistance to fosfomycin (Table 1). They carried 12 to 17 resistance genes. Among them, the β-lactam resistance genes *bla*_TEM-1b_ and *bla*_CTX-M-55_, aminoglycoside resistance genes *aph(3′)-Ia*, *aac(3)-IId*, *aadA17*, and *rmtB*, tetracycline resistance gene *tet*(A), florfenicol exporter *floR*, lincomycin resistance gene *lnu*(F), and rifampin resistance gene *arr-2* were identified in all 10 *S*. Kentucky isolates, whereas the resistance genes *aadA7*, *aac(3)-Iv*, *qnrS1*, *sul1*, *mph*(A), and *fosA3* were detected in 1 to 9 *S*. Kentucky strains (Table 1). One *S*. Infantis isolate and two *S*. Agona isolates also contained multiple resistance genes, resulting in MDR phenotypes (Table 1). In addition, plasmid replicons were identified in 10 *S*. Kentucky strains, whereas no plasmid replicons were detected in *S*. Infantis and *S*. Agona isolates (Table 1).

**TABLE 1:** Characterizations of Salmonella isolates in this study

Strain	Serotype	Source	MLST result	Resistance^*a*^	Resistance genes	Mutation(s) in:		Plasmid replicon(s)
						*gyrA*	*parC*	
AH19MCS1	Kentucky	Drumstick	ST198	AMP, CFZ, CTX, GEN, AMI, STR, TET, TIL, CHL, FFC, NAL, SXT	*bla*_CTX-M-55_, *bla*_TEM-1B_, *aadA7*, *aadA17*, *aph(3′)-Ia*, *aac(3)-IId*, *aac(3)-IV*, *rmtB*, *tet*(A), *sul1*, *dfrA14*, *floR*, *Inu*(F), *mph*(A), *arr-2*, *fosA3*, *qnrS1*	S83F, D87N	S80I	Col156
AH19MCS8	Kentucky	Chicken meat	ST198	AMP, CFZ, CTX, GEN, AMI, STR, TET, TIL, CHL, FFC, NAL, CIP, FOS, SXT	*bla*_CTX-M-55_, *bla*_TEM-1B_, *aadA7*, *aadA17*, *aph(3′)-Ia*, *aac(3)-IId*, *rmtB*, *tet*(A), *sul1*, *dfrA14*, *floR*, *Inu*(F), *arr-2*, *fosA3*	S83F, D87N	S80I	IncI1, Col156, ColpVC, Col440I
AH19MCS11	Kentucky	Chicken feet	ST198	AMP, CFZ, CTX, GEN, AMI, STR, TET, TIL, CHL, FFC, NAL, CIP, SXT	*bla*_CTX-M-55_, *bla*_TEM-1B_, *aadA17*, *aph(3′)-Ia*, *aac(3)-IId*, *rmtB*, *tet*(A), *dfrA14*, *floR*, *Inu*(F), *mph*(A), *arr-2*	S83F, D87N	S80I	IncI-γ, ColpVC, IncN
AH19MCS13	Kentucky	Chicken meat	ST198	AMP, CFZ, CTX, GEN, AMI, STR, TET, TIL, CHL, FFC, NAL, CIP, FOS, SXT	*bla*_CTX-M-55_, *bla*_TEM-1B_, *aadA7*, *aadA17*, *aph(3′)-Ia*, *aac(3)-IId*, *rmtB*, *tet*(A), *sul1*, *dfrA14*, *floR*, *Inu*(F), *mph*(A), *arr-2*, *fosA3*	S83F, D87N	S80I	ColRNAI
AH19MCS3	Kentucky	Chicken liver	ST198	AMP, CFZ, CTX, GEN, AMI, STR, TET, TIL, CHL, FFC, NAL, CIP, FOS, SXT	*bla*_CTX-M-55_, *bla*_TEM-1B_, *aadA7*, *aadA17*, *aph(3′)-Ia*, *aac(3)-IId*, *rmtB*, *tet*(A), *sul1*, *dfrA14*, *floR*, *Inu*(F), *mph*(A), *arr-2*, *fosA3*	S83F, D87N	S80I	Col156
AH19MCS6	Kentucky	Chicken gizzard	ST198	AMP, CFZ, CTX, GEN, AMI, STR, TET, TIL, CHL, FFC, NAL, CIP, FOS, SXT	*bla*_CTX-M-55_, *bla*_TEM-1B_, *aadA7*, *aadA17*, *aph(3′)-Ia*, *aac(3)-IId*, *rmtB*, *tet*(A), *sul1*, *dfrA14*, *floR*, *Inu*(F), *mph*(A), *arr-2*, *fosA3*	S83F, D87N	S80I	Col156, ColRNAI
AH19MCS7	Kentucky	Chicken gizzard	ST198	AMP, CFZ, CTX, GEN, AMI, STR, TET, TIL, CHL, FFC, NAL, CIP, FOS, SXT	*bla*_CTX-M-55_, *bla*_TEM-1B_, *aadA7*, *aadA17*, *aph(3′)-Ia*, *aac(3)-IId*, *rmtB*, *tet*(A), *sul1*, *dfrA14*, *floR*, *Inu*(F), *mph*(A), *arr-2*, *fosA3*	S83F, D87N	S80I	Col156
AH19MCS9	Kentucky	Drumstick	ST198	AMP, CFZ, CTX, GEN, AMI, STR, TET, TIL, CHL, FFC, NAL, CIP, SXT	*bla*_CTX-M-55_, *bla*_TEM-1B_, *aadA17*, *aph(3′)-Ia*, *aac(3)-IId*, *rmtB*, *tet*(A), *dfrA14*, *floR*, *Inu*(F), *mph*(A), *arr-2*	S83F, D87N	S80I	IncI-γ, ColpVC, IncN
AH19MCS10	Kentucky	Drumstick	ST198	AMP, CFZ, CTX, GEN, AMI, STR, TET, TIL, CHL, FFC, NAL, CIP, FOS, SXT	*bla*_CTX-M-55_, *bla*_TEM-1B_, *aadA7*, *aadA17*, *aph(3′)-Ia*, *aac(3)-IId*, *rmtB*, *tet*(A), *sul1*, *dfrA14*, *floR*, *Inu*(F), *mph*(A), *arr-2*, *fosA3*	S83F, D87N	S80I	Col156
AH19MCS12	Kentucky	Chicken feet	ST198	AMP, CFZ, CTX, GEN, AMI, STR, TET, TIL, CHL, FFC, NAL, CIP, FOS, SXT	*bla*_CTX-M-55_, *bla*_TEM-1B_, *aadA7*, *aadA17*, *aph(3′)-Ia*, *aac(3)-IId*, *rmtB*, *tet*(A), *sul1*, *dfrA14*, *floR*, *Inu*(F), *mph*(A), *arr-2*, *fosA3*	S83F, D87N	S80I	Col156
AH19MCS2	Infantis	Chicken wing	ST32	AMP, TET, CHL, NAL, SXT	*bla*_CARB-2_, *aadA1*, *tet*(G), *floR*, *sul1*, *dfrA1*			
AH19MCS4	Agona	Chicken intestine	ST13	AMP, CFZ, CTX, GEN, STR, TET, TIL, CHL, FFC, SXT	*bla*_CTX-M-55_, *aac(3′)-IId*, *aadA1*, *strAB*, *tet*(A), *floR*, *qnrS1*, *fosA7*, *sul3*, *mef*(B), *lnu*(F)			
AH19MCS5	Agona	Chicken lung	ST13	AMP, CFZ, CTX, GEN, STR, TET, TIL, CHL, FFC, NAL, CIP, SXT	*bla*_CTX-M-55_, *aac(3′)-IId*, *aadA1*, *strAB*, *tet*(A), *floR*, *qnrS1*, *fosA7*, *sul3*, *mef*(B), *lnu*(F)			

*^a^*AMP, ampicillin; CFZ, cefazolin; CTX, cefotaxime; GEN, gentamicin; AMI, amikacin; STR, streptomycin; TET, tetracycline; TIL, tigecycline; CHL, chloramphenicol; FFC, florfenicol; NAL, nalidixic acid; CIP, ciprofloxacin; FOS, fosfomycin; SXT, sulfamethoxazole-trimethoprim.

Fluoroquinolones and cephalosporins are the first-line drugs for treating nontyphoidal Salmonella infections (5). *S*. Kentucky ST198 with coresistance to ciprofloxacin and extended-spectrum cephalosporins has been increasingly reported worldwide (8–10, 13–15, 19–21). Plasmid-borne *bla*_CTX-M-1/-15/-25_ or *bla*_CMY_ acquired by *S*. Kentucky is the main reason for cephalosporin resistance (8, 9, 21). Recently, chromosomally located *bla*_CTX-M-14b_ in *S*. Kentucky ST198 has been reported in Europe and China (14, 15, 20). In this study, all *S*. Kentucky ST198 strains contained *bla*_CTX-M-55_, one of the predominant CTX-M genotypes in animals and humans in China (22). The major mechanisms mediating resistance to quinolones are mutations in the quinolone resistance-determining region (QRDR) of *gyrA*, *gyrB*, *parC*, and *parE* and the presence of plasmid-mediated quinolone resistance (PMQR) genes (2). All *S.* Kentucky strains in this study had mutations within *gyrA* (S83F and D87N) and *parC* (S80I) (Table 1), accounting for their high-level resistance to nalidixic acid (MICs of >256 mg/L) and ciprofloxacin (MICs of ≥16 mg/L). A single PMQR gene *qnrS1* or *gyrA* mutation (S83Y) resulted in nalidixic acid resistance only in *S*. Infantis and *S*. Agona strains (Table 1).

Of concern, all *S*. Kentucky and *S*. Agona strains exhibited resistance to tigecycline (MICs of 2 to 8 mg/L), a last-resort antibiotic to treat serious infections caused by MDR bacteria. Mobile tigecycline resistance determinants *tet*(X) and *tmexCD1-toprJ1* or amino acid changes within AcrAB-TolC, RamA, RamR, MarA, and MarR were not identified in 12 tigecycline-resistant strains in this study. We previously confirmed the association of a tetracycline resistance gene *tet*(A) variant with tigecycline resistance in *S*. Kentucky (15). The presence of this *tet*(A) variant in all *S*. Kentucky and *S*. Agona isolates in this study may account for their tigecycline resistance. It further highlights the important role of the *tet*(A) variant in the wide dissemination of tigecycline resistance.

**FIG 1:**
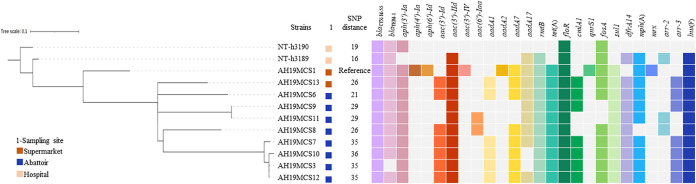
Maximum likelihood tree of *S*. Kentucky ST198 isolates in this study, compared with clinical *S*. Kentucky ST198 isolates (GenBank accession no. PRJNA820366) from China, based on cgSNP analysis.

To further compare the genetic differences among 10 *S*. Kentucky ST198 strains in this study, we performed a phylogenetic analysis based on core genome single-nucleotide polymorphisms (cgSNPs). They showed a close genetic relationship (21 to 36 single-nucleotide polymorphisms [SNPs]), differing from two clinical *S*. Kentucky ST198 strains (GenBank accession no. PRJNA820366) recovered in Nantong, Jiangsu Province, China, in 2021 by only 16 or 19 SNPs (Fig. 1). This finding suggests a potential risk of MDR *S*. Kentucky ST198 transmission to humans along the food chain.

### Location and genetic structures of MRRs in *S*. Kentucky strains.

In order to clarify the genetic structures of numerous resistance genes in *S*. Kentucky, three *S*. Kentucky strains, i.e., AH19MCS1 (supermarket), AH19MCS8 (slaughterhouse), and AH19MCS11 (slaughterhouse), were selected based on their sources and susceptible phenotypes and were further sequenced using Pacific Biosciences (PacBio) single-molecule real-time (SMRT) sequencing. The complete genome sequences of three *S*. Kentucky isolates were obtained (see Table S1 in the supplemental material). In these *S*. Kentucky isolates, all antimicrobial resistance genes were located on their chromosomes and were clustered in one mosaic multiresistance region (MRR) and SGI1-K. No resistance genes were identified on five plasmids in AH19MCS8 and AH19MCS11 (see Table S1).

**FIG 2:**
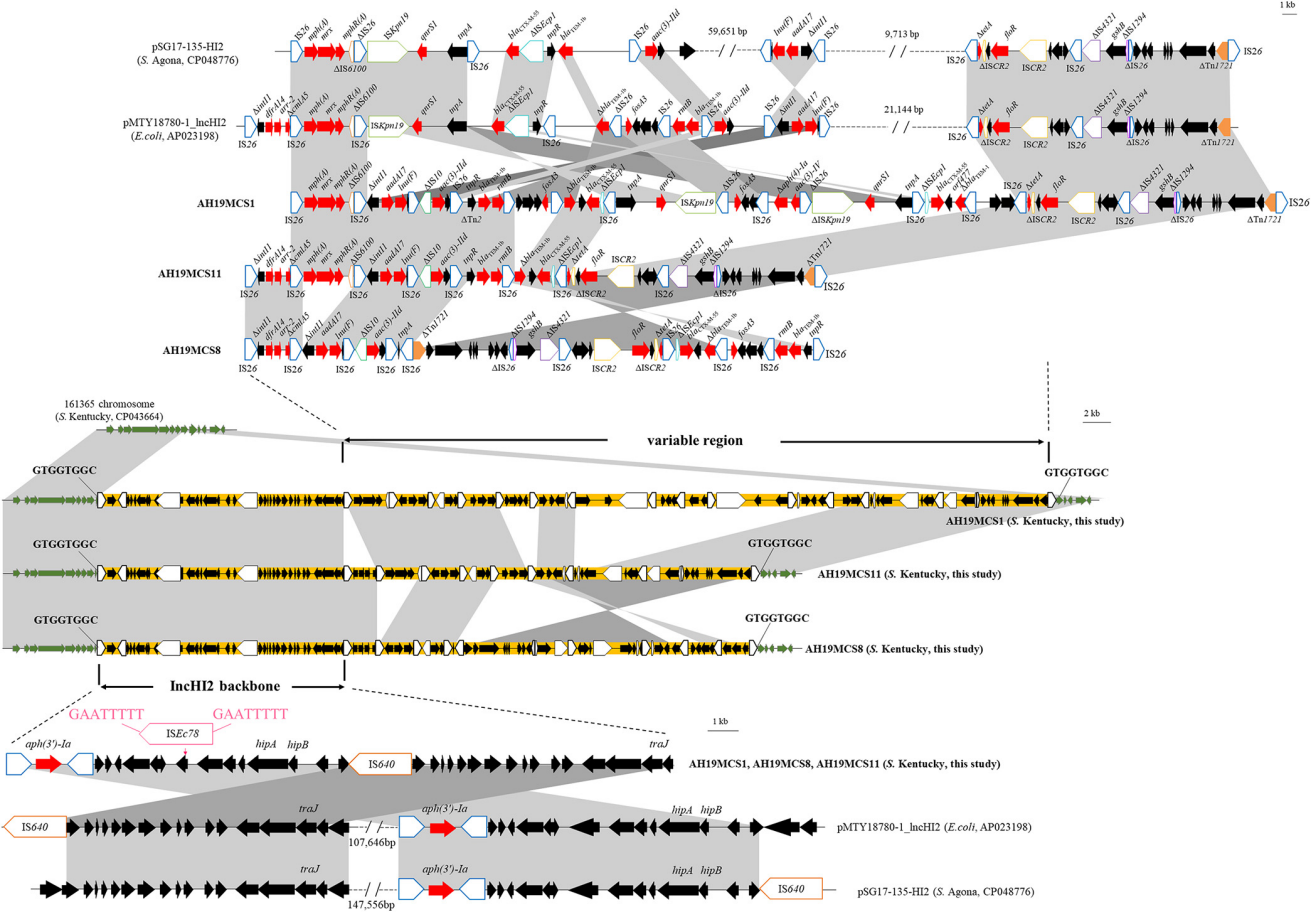
Genetic structure comparison of the MRRs of AH19MCS1, AH19MCS8, and AH19MCS11 with IncHI2 plasmids. Resistance genes are shown with red arrows, genes on the *S*. Kentucky chromosome are shown with green arrows, and other genes are indicated with black arrows. Δ indicates a truncated gene or mobile element. ISs are shown as boxes labeled with their names. Labeled vertical arrows with an IS box indicate the insertion site of an IS element. DRs are indicated by arrows and sequences. Regions with >99.9% identity are shaded in gray.

As shown in Fig. 2, three *S*. Kentucky strains shared similar MRRs. The MRRs were bounded at both ends by IS*26* and inserted into the chromosome with an identical location downstream of the *bcfABCDEFG* cluster encoding fimbrial proteins. The insertion of MRR interrupted a putative DsbA family protein and generated 8-bp direct repeats (DRs) (5′-GTGGTGGC-3′). We further compared the contigs in other *S*. Kentucky strains and found that fragments of IS*26* with identical 8-bp DRs were inserted downstream of the *bcfABCDEFG* cluster in the remaining seven strains, suggesting that similar insertion of MRRs possibly occurred in all *S*. Kentucky strains in this study.

The MRR of AH19MCS1 was the largest (~92.9 kb) and consisted of two parts bounded by IS*26* (Fig. 2). The first part (~23.9 kb) included a 2,820-bp resistance module [IS*26*-*aph(3′)-Ia-*IS*26*] and a variety of open reading frames (ORFs) such as the transcriptional regulators *hipA*/*hipB* and the conjugal transfer gene *traJ*, which was highly (>99.9%) similar to two separate segments corresponding to IncHI2 plasmid backbones, such as pSG17-135-HI2 (*S*. Agona [GenBank accession no. CP048776]) and pMTY18780-1_IncHI2 (Escherichia coli [GenBank accession no. AP023198]), except that one copy of IS*Ec78* was inserted into a putative hypothetical protein with 8-bp DRs (5′-GAATTTTT-3′) in AH19MCS1. The second part (~69.0 kb) contained numerous resistance genes, such as *mph*(A), *aadA17*, *lnu*(F), *aac(3)-IId*, *bla*_TEM-1b_, *rmtB*, *fosA3*, *bla*_CTX-M-55_, *qnrS1*, and *floR*, associated with insertion sequences (ISs), transposons, and integron (e.g., IS*26*, Tn*2*, ΔIS*Ecp1*, IS*Kpn19*, and IS*CR2*).

Compared with that of AH19MCS1, the MRRs of AH19MCS8 and AH19MCS11 differed by the acquisition of a 3,070-bp segment harboring the *dfrA14*/*arr-2*/Δ*cmlA5* cassette array associated with an incomplete class 1 integrase IntI1 truncated by IS*26* and the absence of an ~28.4-kb segment involving the Δ*tnpA*-*qnrS1*-IS*Kpn19*-ΔIS*26*-*fosA3*-IS*26*-Δ*aph(4)-Ia*-*aac(3)-Ia*-ΔIS*26*-ΔIS*Kpn19*-*qnrS1*-ΔIS*Ecp1*-*bla*_CTX-M-55_-Δ*bla*_TEM-1b_-IS*26-*IS*26* structure (Fig. 2). Additional deletion of a 3,317-bp *fosA3* resistance module (*fosA3*-*orf1*-*orf2*-Δ*orf3*-IS*26*) or a 4,373-bp macrolide phosphotransferase region [IS*26*-*mph*(A)-*mrx*-*mphR*(A)-ΔIS*6100*] was observed in AH19MCS11 or AH19MCS8, respectively, and an approximately 29.5-kb segment (IS*26*-ΔTn*1721*-*gshB*-ΔIS*4321*-IS*26*-IS*CR2*-*floR*-Δ*tetA*-IS*26*-IS*Ecp1*-*bla*_CTX-M-55_-Δ*bla*_TEM-1b_-IS*26*-*fosA3*-IS*26*-*rmtB*-*bla*_TEM-1b_-IS*26*) was in the opposite location in the latter one (Fig. 2). IS*26*-mediated homologous recombination could readily explain these differences.

The MRRs of AH19MCS1, AH19MCS8, and AH19MCS11 were related to those of IncHI2 plasmids pSG17-135-HI2 and pMTY18780-1_IncHI2 but differed by insertions, deletions, and rearrangement of multiple segments involving resistance genes and plasmid backbones, which were possibly mediated by mobile elements such as IS*26* and IS*640* (Fig. 2). This finding indicates that the MRR fragment inserted into chromosomes of *S*. Kentucky isolates is possibly acquired from IncHI2 plasmids. Previously, additional resistance genes outside the SGI, such as *bla*_CTX-M_, *bla*_OXA-48_, *bla*_CMY-2_, *bla*_VIM-2_, and *mph*(A), in *S*. Kentucky ST198 were likely carried by plasmids except for *bla*_CTX-M-14b_ (8, 9, 20). An IncHI2 plasmid carrying numerous resistance genes was previously described for one *S*. Kentucky ST198 isolate from chicken in China (10). However, numerous resistance genes are clustered in the chromosomes of *S*. Kentucky ST198 strains in this study, enabling them to transfer vertically as intrinsic chromosomal genes within this lineage. More importantly, the strains are capable of acquiring more resistance genes with the help of mobile elements through the process of dissemination, resulting in the emergence of extensively drug-resistant *S*. Kentucky strains.

### Structures of SGI1-K detected in *S*. Kentucky strains.

**FIG 3:**
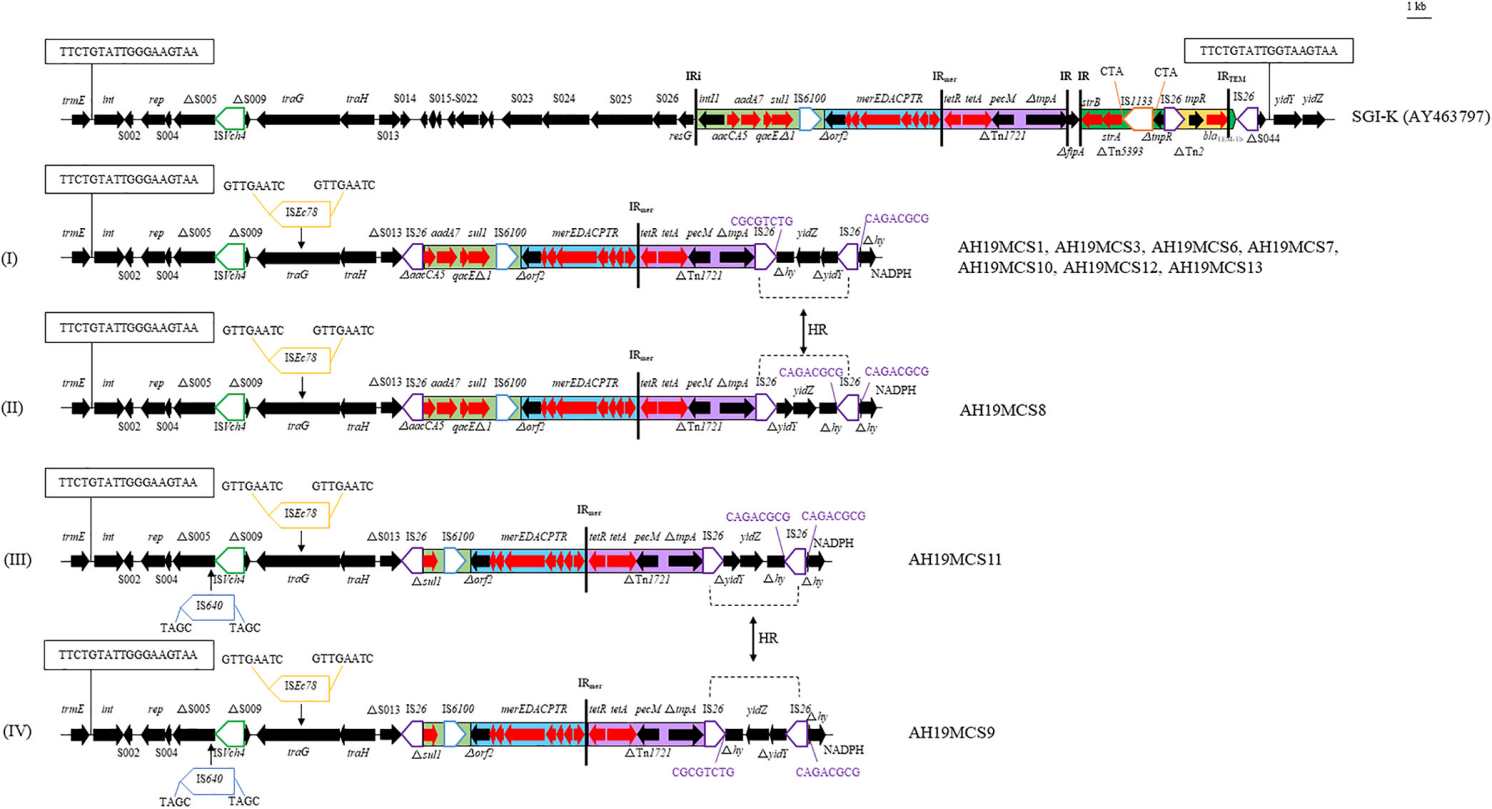
Genetic structures of SGI1-K variants of *S.* Kentucky strains in this study, compared with SGI1-K. Resistance genes are shown with red arrows, and other genes are indicated with black arrows. Δ indicates a truncated gene or mobile element. ISs are shown as boxes labeled with their names. Labeled vertical arrows with an IS box indicate the insertion site of an IS element. DRs are indicated by arrows and sequences. Tall bars represent the inverted repeats (IRs) of a transposon or integron. Arrows labeled HR and dashed lines indicate where homologous recombination could explain differences between structures.

Four SGI1-K variants (types I to IV) with slight differences were identified in 10 *S*. Kentucky strains in this study, with type I (*n* = 7) being the most common type. As previously described for numerous *S*. Kentucky ST198 strains (6, 9, 14, 15), SGI1-K variants were also integrated into the 3′ end of the chromosomal *trmE* gene in our *S.* Kentucky strains (Fig. 3). Compared with the prototype of SGI1-K (GenBank accession no. AY463797), one copy of IS*Ec78* was inserted into backbone gene *traG* and generated 8-bp DRs in four SGI1-K variants, and an additional insertion of IS*640* into S005 flanked by 4-bp DRs was observed in AH19MCS9 and AH19MCS11 (Fig. 3).

The MRRs of four SGI1-K variants were bounded by two copies of IS*26* with opposite orientations. The insertion of MRR resulted in the absence of large backbone segments, including S013 (13 bp), S014 to S026, and *resG*, as well as ΔS044, the downstream 18-bp recombination site duplication, and 524 bp of the 5′ end of *yidY*, compared with SGI1-K (Fig. 3). The MRRs of SGI1-K variants were identical in eight strains (type I and type II). They lacked an 8,850-bp segment involving partial class 1 integron (*intI1*-Δ*aacCA5*), 417-bp transposon Tn*1721*, Δ*fipA*, core SGI1-K resistance genes *strA* and *strB* within Tn*5393*, and *bla*_TEM-1b_ within Tn*2*, and IS*26*, compared with SGI1-K. An additional deletion of a 2,065-bp segment (Δ*aacCA5-aadA7-qacE*Δ*1-*Δ*sul1*) was observed in AH19MCS9 and AH19MCS11.

In addition, one copy of IS*26* was inserted into one hypothetical protein downstream of the *yidZ* gene in AH19MCS8 and AH19MCS11, with 8-bp DRs. Homologous recombination between this IS*26* element and the upstream IS*26* with opposite orientation could explain the inversion of the 2,426-bp intervening segment (Δ*yidY*-*yidZ-*Δ*hy*) observed in the remaining eight *S*. Kentucky strains (Fig. 3).

To date, many SGI1-K variants in *S.* Kentucky ST198 have been reported globally; their diverse structures indicate that they might undergo rapid evolution within this clone, mainly due to insertions, deletions, or rearrangements of backbone segments or resistance modules mediated by IS*26* (9, 10, 14, 15). SGI1 carrying multiple resistance genes has been found in chromosomes of diverse S. enterica serovars, Proteus mirabilis, Klebsiella pneumonia, Escherichia coli, and some other species (7, 23–25) and could be mobilized with the help of IncA/C plasmids (7). The global spread of strains containing SGI1, such as *S*. Kentucky ST198 here, should be paid more attention for their important roles in the dissemination of resistance genes.

### Conclusion.

In this study, we investigate the genomic characterization of extensively drug-resistant *S.* Kentucky ST198 strains from chicken meat products from a city in China. All resistance genes are located in the chromosomes of *S*. Kentucky, clustered in a SGI1-K structure or MRR that possibly originated from IncHI2 plasmid. Mobile elements, particularly IS*26*, play an important role in generating diverse resistance regions within chromosomes. The emergence of extensively drug-resistant *S*. Kentucky ST198 strains carrying numerous chromosomally located resistance genes in this study is worrisome. Their resistance to many clinically critical antimicrobial agents limits clinical choices for infection treatments, and the dissemination of these *S.* Kentucky strains will facilitate the dissemination of numerous resistance genes. Therefore, continuous surveillance needs to be performed to monitor this high-risk clone and antimicrobial resistance in animals and animal-derived food products, and appropriate measures, such as regular full-scale disinfection in the production chain and the application of antibiotic alternatives in farms, should be taken to minimize contamination.

## MATERIALS AND METHODS

### Sample collection and Salmonella isolation.

On 8 December 2019, 75 samples of chicken meat (*n* = 59) and chicken internal organs (*n* = 16) were collected from two supermarkets and a slaughterhouse in Xuancheng, Anhui Province, China. The samples were incubated in 100 mL buffered peptone water (BPW) for 18 to 24 h at 37°C with constant shaking at 180 rpm. Then, the enriched 1-mL BPW cultures were transferred to 10 mL Rappaport-Vassiliadis R10 broth (RVR10) and incubated for 24 h at 42°C. The cultures were further inoculated onto XLT4 agar plates and incubated for 24 h at 37°C. One suspicious isolate per plate was purified and confirmed by detection of the *stn* gene for Salmonella identification using PCR and sequencing (26).

### Antimicrobial susceptibility testing.

The antimicrobial agent MICs of Salmonella strains were determined by using the agar dilution method or the broth microdilution method (limited to colistin and tigecycline). The following antimicrobial agents were tested: ampicillin, cefazolin, cefotaxime, meropenem, gentamicin, streptomycin, amikacin, tetracycline, tigecycline, chloramphenicol, florfenicol, nalidixic acid, ciprofloxacin, colistin, fosfomycin, and sulfamethoxazole-trimethoprim. E. coli ATCC 25922 was used as the quality control strain. The results were interpreted according to EUCAST clinical breakpoints (https://www.eucast.org/clinical_breakpoints) and ECOFF (https://www.eucast.org/mic_and_zone_distributions_and_ecoffs).

### Whole-genome sequencing and analysis.

Bacterial DNA was extracted using the TIANamp bacteria DNA kit (Tiangen, Beijing, China). All Salmonella isolates were sequenced on the Illumina HiSeq platform. The raw reads were assembled into contigs with SPAdes v.3.8.2. The serotypes were analyzed by the Salmonella *In Silico* Typing Resource (SISTR) (27). Three *S.* Kentucky strains, i.e., AH19MCS1 from a supermarket and AH19MCS8 and AH19MCS11 from a slaughterhouse, were selected as representatives and were sequenced using PacBio SMRT sequencing technology. The genomic sequences were further analyzed by multilocus sequence typing (MLST) and analysis of chromosomal mutations, resistance genes, and plasmid replicons with the Center for Genomic Epidemiology (CGE) platform (http://www.genomicepidemiology.org). The SGI and MRR structures in AH19MCS1, AH19MCS8, and AH19MCS11 were analyzed by the RAST server (28), ISfinder (https://www-is.biotoul.fr), and BLAST (https://blast.ncbi.nlm.nih.gov/Blast.cgi). Resistance genes and plasmid replicons were identified with >90% sequence homology and coverage. Contigs carrying SGI1-K fragments were extracted and assembled by PCR and sequencing (see Table S2 in the supplemental material) using SGI1-K variants detected in isolates AH19MCS1, AH19MCS8, and AH19MCS11 as references. The phylogenetic tree of 10 *S*. Kentucky strains in this study and 2 *S*. Kentucky strains from patients in China (29) was constructed using Parsnp (https://harvest.readthedocs.io/en/latest/content/parsnp.html) and visualized with iTOL (30).

### Data availability.

The whole-genome sequences of all Salmonella isolates have been deposited in GenBank under accession no. PRJNA868865.
