# Supplementary material for: Characterization of Extensively Drug-Resistant Salmonella enterica Serovar Kentucky Sequence Type 198 Isolates from Chicken Meat Products in Xuancheng, China
Source: Microbiol Spectr. 2023 Feb 27;11(2):e03219-22. doi: 10.1128/spectrum.03219-22 (PMC10100706; doi:10.1128/spectrum.03219-22)
Supplement: Supplemental file 1 — Table S1. Download spectrum.03219-22-s0001.pdf, PDF file, 0.4 MB [file spectrum.03219-22-s0001.pdf]

Table S1 Complete genome sequences of three *S.* Kentucky ST198 isolates in this study.

	Size(bp)	Resistance genes	Plasmid replicons
**AH19MCS1**			
chromosome	4,948,090	*bla*$_{CTX-M-55}$/*bla*$_{TEM-1B}$/*aadA7*/*aadA17*/*aph(3')-Ia/aac(3)-IId* /*aac(3)-IV/rmtB*/*tet*(A)/*sul1*/*dfrA14/floR*/*Inu*(F)/*mph*(A)/*arr-2/* *fosA3*/*qnrS1*	
**AH19MCS8**			
chromosome	4,882,259	*bla*$_{CTX-M-55}$/*bla*$_{TEM-1B}$/*aadA7*/*aadA17*/*aph(3')-Ia/aac(3)-IId/* *rmtB*/*tet*(A)/*sul1*/*dfrA14/floR*/*Inu*(F)/*arr-2/ fosA3*	
pYUAHMCS8-1	85,309	none	IncI1
pYUAHMCS8-2	4,234	none	/
pYUAHMCS8-3	4,197	none	Col156
pYUAHMCS8-4	3,428	none	/
pYUAHMCS8-5	3,374	none	/
**AH19MCS11**			
chromosome	4,905,193	*bla*$_{CTX-M-55}$/*bla*$_{TEM-1B}$/*aadA17*/*aph(3')-Ia/aac(3)-IId* /*rmtB*/*tet*(A)/*dfrA14/floR*/*Inu*(F)/*mph*(A)/*arr-2*	
pYUAHMCS11-1	8,4242	none	IncI-γ, IncN
pYUAHMCS11-2	6,912	none	/
pYUAHMCS11-3	4,657	none	/
pYUAHMCS11-4	3,373	none	/
pYUAHMCS11-5	2,058	none	ColpVC

Table S2 Primers used to assemble SGI1-K variants in this study

Region	Nucleotide Sequence (5' to 3')	Size (bp)
S005-IS*Vch4*-S009-*traG*	F:CTGTCCAGCAGTCCA<br>R: GCAGGGTTGTATGTGA	1702
traG-IS*Ec78*-*traG*	F: GATTAGCGGCATTTAC<br>R: GGACCGTGGTTCTGT	2894
S013-IS*26*-*aacA5*	F: GCCGCTAATAACACGA<br>R: CTCTATGGCTGGTTGG	1327
S013-IS*26*-*sul1*	F: GCCGCTAATAACACGA<br>R: CGACACCGAGACCAAT	1347
Tn*1721*-IS*26*-*yidY*	F: TGTCCGCAATCCTCG<br>R: CGCCACGGCTAAATC	1769
yidZ-hy-IS*26*-NADPH	F: AACGATCCCAACGAA<br>R: TTGCGAATCTGCTCC	1414
Tn*1721*-IS*26*-*yidZ*	F: TTCCACCTATGTGCTCG<br>R: CACCCTTTCTCCCGTAA	1531
yidY-IS*26*-NADPH	F: ACCAACCGAGAATCCG<br>R: TCCCAGCAATGTAACCAC	1366
